# If it doesn’t have a soul, it doesn’t hurt? The effect of bullying and social anxiety on the use of conversational AI

**DOI:** 10.3389/fpsyg.2026.1867609

**Published:** 2026-10-23

**Authors:** Gergő Pál, Andras Norbert Zsido, Julia Basler

**Affiliations:** 1 Institute of Psychology, University of Pécs, Pécs, Hungary; 2 Research Centre for Contemporary Challenges, University of Pécs, Pécs, Hungary

**Keywords:** anger displacement, artificial intelligence, bullying, cyberbullying, social anxiety

## Abstract

**Introduction:**

Conversational artificial intelligence (CAI) increasingly functions as a social interaction partner, raising questions about how established interpersonal behaviors transfer to human-AI interaction. The present study examined whether bullying-like behavior directed toward CAI reflects the extension of real world social strategies, and how social anxiety, prior bullying experiences, and AI self-competency contribute to such behavior.

**Methods:**

Participants (*N* = 289) completed measures of social anxiety, prior bullying perpetration and victimization, AI self-competency, and bullying-like behavior directed toward conversational AI.

**Results:**

Through several analyses, bully-victims exhibited the highest levels of AI-directed bullying-like behavior. The interaction between bullying perpetration and victimization emerged as the strongest predictor in our regression analysis, while prior bullying perpetration alone was not sufficient to explain AI-directed aggression, and prior victimization alone was negatively associated with AI-directed bullying-like behavior. Social anxiety did not directly predict bullying-like behavior toward AI; however, mediation analyses showed that prior victimization fully mediated the relationship between social anxiety and bullying-like behavior towards CAI. Individuals with higher social anxiety were more likely to report victimization, which in turn was associated with increased bullying-like behavior toward AI. AI self-competency did not emerge as an independent predictor.

**Discussion:**

These findings suggest that AI-directed bullying-like behavior is best understood as a context-dependent behavior arising from the co-occurrence of aggression and vulnerability, rather than a direct extension of general aggressive tendencies. Conversational AI constitutes a distinct relational context that can both reflect and reinforce existing behavioral patterns. This has important implications for understanding how human-AI interactions may shape social behavior and psychological functioning over time.

## 1 Introduction

Interactions with technologies that can understand, process and respond to human language in a natural, human-like way take an ever-growing place in everyday life and extend beyond instrumental use and increasingly resemble social exchanges. These are called conversational artificial intelligence (CAI). Importantly, CAI encompasses a heterogeneous group of systems that differ in modality (e.g., text-based chatbots, voice assistants, embodied agents). The present paper primarily focuses on text-based conversational interactions, which currently represent the most widespread form of user-AI engagement and are typically mediated by chatbot-style interfaces, often powered by large language models. Due to their linguistic fluency, responsiveness, and conversational turn-taking, users frequently respond to newer AI systems in anthropomorphic ways, attributing agency, intentionality, and social roles to artificial agents (Kim and Im, 2023; Sundar, 2020).

From an evolutionary perspective, interactions with CAI may constitute as an evolutionary mismatch. Such mechanisms originally evolved to solve recurring adaptive problems in ancestral environments (Cosmides and Tooby, 1994) are activated by evolutionarily novel stimuli (Li et al., 2018; Lim et al., 2026). One particularly relevant mechanism is agency detection, which biases individuals to perceive intentional agents in ambiguous contexts. While this tendency was adaptive in environments where failing to detect agents carried high costs, it may also lead to false positives, especially in modern settings involving artificial systems. In parallel, theory of mind allows humans to infer the mental states of others, and shared intentionality, as described by Tomasello and Carpenter (2007), enables coordinated social interactions through the alignment of goals and intentions. Importantly, these attributions occur implicitly and do not depend on explicit beliefs that the system is conscious, or human, since they seem to be driven more by the perceiver’s own prior beliefs and expectations, rather than a system’s actual design (Hortensius and Cross, 2018). This is also supported by the increasing number of research indicating that the rapid development of AI necessitates integrated frameworks within human-AI interactions (Alqasir, 2025; Bigman et al., 2026; Boyd and Markowitz, 2026; Sundar, 2020).

The emerging field of AI Psychology (Alqasir, 2025) suggests that AI is not only a technology used for transactional interactions anymore, but users’ shift toward sustained social engagement with it, and its influence on human cognition, social interactions, and even emotions, require to view AI as a ‘relational partner’. This parallels arguments in human-robot interaction proposing that personified artificial agents may constitute an entirely new ontological category, one that resists classification along the traditional living/nonliving or person/thing dichotomy (Gunkel, 2023; Kahn et al., 2011; Severson and Carlson, 2010; Weisman, 2022). Interestingly, unlike judgments of humans, negative judgments of one AI system’s actions seem to generalize to perceptions of AI as a whole, suggesting that people treat AI as a less individuated, more homogeneous kind of social agent (Manoli et al., 2025). As a separate field within psychology, the core of AI Psychology is the need to understand human-AI dynamics like anthropomorphism, emotional responses, and use behavior. As Guingrich and Graziano (2024) argue, what matters in this context is not whether AI is truly conscious, but whether it is *perceived* as conscious by the human interacting with it, as this perception is sufficient to activate social-cognitive schemas similar to those engaged during human-human interaction (i.e., a carry-over effect). A comparable mechanism is described by the Computers Are Social Actors (Nass et al., 1994) paradigm, which holds that people apply social scripts developed for human interaction to computers and other artificial agents (Gambino et al., 2020).

Chatbots already have a number of purposes such as providing social and emotional support, rather than just gathering information or providing entertainment (Brandtzaeg and Følstad, 2017), which can also be extended to CAI use (Karnaze and Bloss, 2026). Voice assistant AI, social robots and chatbots are endowed with human-like voices, and have the potential to provide homophily with the user, which in turn leads to greater social engagement (Sundar, 2020), and even pseudo-social relationships (Chu et al., 2025; Hu et al., 2023; Manoli et al., 2026; Pentina et al., 2023). This is further supported by findings that anthropomorphism is especially strong when the nonhuman entity possesses traits or features that resemble humans (Epley et al., 2007; Lim et al., 2026; Maeda, 2025). The perception of CAI as a social agent seems to activate the same underlying motivational systems associated with affiliation, self-protection (i.e., avoidance), and status (Hortensius and Cross, 2018; Li et al., 2018; Lim et al., 2026), and can even alleviate loneliness (De Freitas et al., 2026).

Other than positive effects, interacting with AI can also evoke strong emotional responses such as frustration, irritation, or loss of control. This is especially true when systems behave unpredictably or fail to meet expectations. Individual differences in how well people understand, interpret, and regulate their interactions with AI systems are commonly conceptualized under the umbrella of AI literacy (Long and Magerko, 2020). AI literacy refers not only to technical knowledge about how AI systems function, but also to users’ capacity to manage AI-mediated information, influence, and emotional responses. While many approaches to this exist, Carolus et al. (2023a) argue that AI self-competency is a central psychological foundation, defined as the subjective ability to manage one’s behavior, emotions, and agency when interacting with AI systems. From this perspective, effective AI interaction requires not only knowing how to use AI, but also being able to regulate emotional reactions that arise during use and to maintain personal control over AI-supported decisions. Emotion regulation is particularly important, as negative emotional arousal can impair adaptive interaction and reduce perceived control, fostering reactive or maladaptive responses (Carolus et al., 2023a). At the same time, AI self-competency involves recognizing and limiting AI’s influence on everyday decision-making, especially in domains with personal relevance. As AI systems increasingly offer recommendations, guidance, and feedback, preserving user agency depends on the ability to critically evaluate AI input and decide when to rely on, resist, or override it. Together, these aspects shape how individuals emotionally and behaviorally respond to conversational AI (Carolus et al., 2023b).

Differences in social behavior toward CAI as a social agent need to be investigated, since interpersonal strategies that were calibrated for human-human interactions throughout human evolution may be (mis)applied to artificial agents, as shown in previous research (Chu et al., 2025; De Freitas et al., 2026; Kirk et al., 2025; Morgante et al., 2024). CAI presents a unique context in which such strategies can manifest without the constraints of reputational consequences. As conversational agents might not be able to experience harm, rejection, or emotional distress, users may exhibit dominance-related social strategies such as verbal abuse, name-calling, or threats toward them – behaviors that, when expressed toward another human, are parts of *bullying* behavior. It is important to note that although CAI does not constitute a traditional victim, repeated verbal abuse, domination attempts, and demeaning language toward an artificial agent can be conceptualized as bullying-like behavior insofar as they reflect dominance assertion toward a perceived social target. While bullying can be viewed as a strategy evolved for managing social hierarchy and evaluation (Volk et al., 2022), it is important to examine whether it also manifests toward CAI in order to navigate the novelty of technological advancement, as bullying-like behavior toward an artificial agent might not simply be a low-cost rehearsal of human-human aggression, but a behavior intrinsic to the evolving human-AI relational system (Boyd and Markowitz, 2026). Importantly, we do not assume full equivalence between bullying directed toward humans and behavior directed toward artificial agents. Traditional definitions of bullying involve components such as harm, power imbalance, and social consequences, which are – at the point of data collection – not applicable to AI in the same way as to humans. In the present study, we are referring to bullying-like behavior toward AI as the expression of dominance-oriented and verbally aggressive actions toward a perceived social target that elicits social-cognitive responses. To our best knowledge the number of studies on the field remains scarce, as previous studies only examined CAI as a way to prevent it (i.e., Ige and Adewale, 2022) and none to date has examined bullying in the context of abuse directed toward the AI itself.

In this context, the results of bullying research on bully-victims, who both experience victimization and engage in bullying behavior, might be important. These individuals show higher levels of psychological distress, including anxiety, emotional dysregulation, and maladaptive coping strategies (Arató et al., 2020; Coelho and Romão, 2018). In cyber contexts specifically, bully-victims tend to display higher levels of moral disengagement and difficulties in emotion regulation compared to uninvolved individuals, indicating that aggressive behavior may function as a response to unresolved distress following victimization rather than as a primary trait (Arató et al., 2020). This pattern supports the view that prior experiences of being targeted may increase the likelihood of later aggressive or domineering behavior, particularly in contexts where direct retaliation is possible without substantial social risk, like in a conversation with an AI system.

Social anxiety (oriented toward threat avoidance and evolved to protect from social exclusion and status loss; Heimberg et al., 2014) may both precede and result from bullying involvement. Social anxiety contributes to a maladaptive cycle in which anxiety heightens vulnerability to victimization, while the victimization reinforces defensive or aggressive responses (Pabian and Vandebosch, 2021; Siegel et al., 2009). Previous research indicates that bully-victims often exhibit high levels of social anxiety and social withdrawal (Coelho and Romão, 2018; Juvonen et al., 2003; Swearer et al., 2001). Further, socially anxious individuals often engage more readily in online communication than face-to-face interaction, as the digital environment allows for greater control over self-preservation and reduced fear of immediate negative evaluation (Erwin et al., 2004; Navarro et al., 2015). Social anxiety has also been linked to the Problematic Use of CAI; Hu et al.’s 2023) research proposed that AI might extricate individuals with social anxiety from the discomfort of the interpersonal interactions with real humans through pseudo-social interactions.

Some individuals even engage in online aggression either as a safer outlet of frustration and anger, or as a form of displacement (Martin and Vieaux, 2015). The combination of heightened anxiety, prior victimization, and reduced interpersonal risk might mean that CAI may provide a uniquely low-risk outlet for dominance-related behaviors among socially anxious individuals with a history of victimization. CAI systems present social cues sufficient to activate social-evaluation and dominance-tracking mechanisms but can be perceived to lack genuine reciprocity, reputational memory, or the ability to retaliate. For individuals who are highly sensitive to social threat yet motivated to regain control, this may create a context in which bullying behavior can be expressed without fear of negative evaluation or punishment.

The present study aims to examine how established interpersonal social strategies, particularly bullying and anxiety-related processes, extend to interactions with conversational AI. *First, since bully-victims might have shown increased moral disengagement in cyber-contexts, we hypothesized that they will show the highest levels of bullying-like behavior toward CAI, as compared to individuals who are not in the bully-victim status (H1)*. Second, considering that bullying reflects a generalized dominance-oriented interpersonal strategy that can be expressed toward perceived social agents, and individual differences in AI self-competency are expected to shape behaviors toward it, we hypothesize that *higher levels of social anxiety, greater prior involvement in bullying (as perpetrator as well as victim), and lower levels of AI self-competency are expected to contribute to increased bullying-like behavior toward CAI (H2)*. Third, given evidence that bullying victimization and social anxiety are tightly intertwined, and that victimization may foster defensive or displaced aggression in low-risk contexts, we propose that *prior victimization mediates the relationship between social anxiety and bullying-like behavior toward AI, such that higher social anxiety is associated with greater victimization experiences, which in turn increase the likelihood of bullying-like behavior toward AI (H3)*.

## 2 Methods

### 2.1 Participants and procedure

To ensure adequate statistical power we determined the required sample size using G*Power 3 (Faul et al., 2007) with a medium effect size (*f* = 0.15), power of 0.8 or greater, and six predictors, yielding a minimum of 98 participants. We deliberately targeted a larger sample size to ensure robustness in both the regression and mediation analyses. We employed a cross-sectional quantitative design using online self-report questionnaires. A total of 289 participants between 18 and 62 years of age (*M* = 22.86; SD = 6.18) completed the study. Table 1 shows detailed descriptive statistics of the sample including age, biological sex, education level, ethnicity, and place of residence.

**Table 1:** Descriptive statistics of the sample.

Variable	Category	*n*	%
Sex	Female	208	72.0
	Male	76	26.3
	Prefer not to say	5	1.7
Education	High school diploma	198	68.5
	Bachelor’s degree	73	25.3
	Master’s degree	16	5.5
	PhD or higher	2	0.7
Ethnicity	Caucasian	199	68.9
	Middle Eastern / North African	44	15.2
	Asian	27	9.3
	Other	19	6.6
Place of residence	Village	26	9.0
	Small town	44	15.2
	City	159	55.0
	Capital	60	20.8

Data collection was conducted through a Google Forms questionnaire in November to December of 2024. Invitations to participate were posted on various forums and mailing lists, and all participants were recruited via the internet. Participants first completed an informed consent form, after which they were asked to provide demographic information. This was followed by questions assessing the frequency and type of CAI tool usage measured on a 5-point Likert scale ranging from Never to Almost every day. The measured tools included ChatGPT, Character.AI is one expression, Microsoft Copilot, Gemini, Amazon Lex, Customer-Service-related chatbots and a category for other CAI (see Table 2 for descriptives). Participants then completed the self-report questionnaires. The study was sent out to various social media platforms on the internet (Reddit, Facebook, Instagram), peers and interested parties, and took approximately 15 to 20 min to complete. Only fully completed surveys were stored and analyzed; incomplete responses were excluded from the analysis. The research was approved by the (redacted for peer review), and was conducted in accordance with the World Medical Association’s Code of Ethics (Declaration of Helsinki). Respondents participated voluntarily and answered the survey online. Written informed consent was obtained from all participants.

**Table 2:** AI use frequency reported by participants.

Never	Once every few months	A couple times a month	Once or twice a week	(Almost) Every day
ChatGPT	21 (7.3%)	27 (9.3%)	54 (18.7%)	68 (23.5%)
Character.AI	227 (78.5%)	24 (8.3%)	18 (6.2%)	10 (3.5%)
Microsoft Copilot	218 (75.4%)	31 (10.7%)	17 (5.9%)	12 (4.2%)
Google Gemini	193 (66.8%)	35 (12.1%)	26 (9.0%)	23 (8.0%)
Amazon Lex	263 (91.0%)	11 (3.8%)	10 (3.5%)	3 (1.0%)
Customer service-related	179 (61.9%)	64 (22.1%)	31 (10.7%)	14 (4.8%)
Other	197 (68.2%)	38 (13.1%)	27 (9.3%)	16 (5.5%)

### 2.2 Questionnaires

We used self-report questionnaires to assess past experiences of bullying and victimhood, previous knowledge about artificial intelligence, and the social anxiety levels of participants. The descriptive statistics of all questionnaires can be found in Table 3; we computed scores by summing the relevant items for each subscale.

**Table 3:** Descriptive statistics of the used scales.

Variable	Min	Max	*M*	SD	Skewness	Kurtosis
BCBQ bullying	8	32	11.98	4.90	1.85	3.06
BCBQ victimization	8	40	16.26	6.63	0.89	0.56
BCBQ AI bullying	6	22	7.86	3.77	2.16	3.53
MAILS apply AI	6	30	20.31	7.11	−0.34	−0.89
MAILS persuasion lit.	3	15	11.72	2.94	−0.70	−0.09
MAILS emotion reg.	3	15	11.45	3.02	−0.57	−0.25
SIAS	0	24	7.60	5.29	0.59	−0.31

BCBQ, Bullying and Cyberbullying Behaviors Questionnaire – Short Form; MAILS, Meta AI Literacy Scale; SIAS, Social Interaction Anxiety Scale – Short Form.

Social anxiety was measured using the Short Form of the Social Interaction Anxiety Scale (SIAS-6) (Peters et al., 2012), comprising six items on a 4-point Likert-scale ranging from 0 (not at all a characteristic of me) to 4 (extremely characteristic of me). Higher scores indicate higher levels of interaction-related anxiety. The questionnaire demonstrated good internal consistency (ω = 0.839).

The Bullying and Cyberbullying Behaviors Questionnaire-Short Form (BCBQ-SF; Coelho and Sousa, 2020) consists of 16 items, loading onto 2 factors: Bullying (8 items; ω = 0.862), and Victimization (8 items; ω = 0.866). For the purpose of the present study, the BCBQ-SF was also modified to assess bullying-like behaviors directed toward Conversational AI. Six items were rephrased to refer specifically to interactions with CAI while the original behavioral content was retained, following the guidelines of the International Test Commission (2018) adapted to this study. Two independent researchers reviewed and reworded the items, after which a panel of expert psychologists (including the two item-reviewers, a PhD student and a senior researcher) worked out the final phrasing of the items and which ones to retain. Before data collection, a pilot study with 13 students from our research group was conducted to assess face validity and correct any issues. As no issues were noted, the scale was used in the study (see items in Table 4). As we aimed to retain the original behavioral content, we excluded items that described behaviors not applicable to AI, e.g., physical harm or social exclusion. The assessed interactions predominantly involved text-based conversational interfaces. While the original BCBQ-SF measures interpersonal bullying, the adapted version used in this study targets the expression of analogous behaviors toward conversational AI. These items capture core behavioral components of bullying (e.g., verbal aggression, threats, teasing), independent of whether the target can experience harm. Therefore, the BCBQ-AI should be interpreted as assessing bullying-like behavioral tendencies rather than bullying in the strict interpersonal sense. The resulting BCBQ-AI subscale had high internal consistency (ω = 0.896). A confirmatory factor analysis confirmed that the one factor fit is suitable (CFI = 0.980, TLI = 0.967, SRMR = 0.025, RMSEA = 0.083 CI [0.04–0.12], χ^2^/df = 2.95). For both the BCBQ-SF and the BCBQ-AI, participants had to respond on 5-point Likert scales ranging from 1 (never) to 5 (always).

**Table 4:** The modified version of the BCBQ-SF.

BCBQ-SF original	BCBQ-SF CAI
I sent threatening or rude messages via Internet.	I sent threatening or rude messages to Conversational AI.
I groped, made sexually offensive gestures or comments toward people.	I made sexually offensive gestures or comments to Conversational AI.
I deliberately tried to frighten or threaten someone.	I deliberately tried to frighten or threaten Conversational AI.
I called people mean names.	I called Conversational AI names.
I made fun of or teased people.	I teased Conversational AI or made fun of them.
I told someone I would not like them unless they did what I said.	I told Conversational AI, I would not like them unless they did what I said.

Responses were gathered on a 5-point Likert-scale (ranging from never to always). Participants were asked to respond how often during their conversations with CAI they used the mentioned behaviors.

Participants’ familiarity with artificial intelligence was measured using the Meta AI literacy scale (MAILS; Carolus et al., 2023b), which originally consists of 34 items gathered into nine factors. We used three subscales with a total of 12 items, which participants had to rate on 5-point Likert scales ranging from 1 (not true at all) to 5 (completely true). The Apply AI subscale (6 items) measures the knowledge of basic functions of AI, and how well an individual can apply it to different scenarios. The AI Persuasion literacy subscale (3 items) measures whether the individual is able to control AI’s influence on oneself, with higher scores reflecting a greater level of self-competency. The AI Emotion regulation subscale (3 items) focuses on the control of both positive and negative emotions when using AI, with a higher score reflecting higher control ability. The internal consistency for the Apply AI scale was excellent (ω = 0.942), for the AI Persuasion literacy was close to the commonly accepted threshold for adequacy (ω = 0.699), and for the AI Emotion regulation was acceptable (ω = 0.738).

### 2.3 Statistical analysis plan

We used skewness and kurtosis values to determine if the data were normally distributed (see Table 3). The Bullying and AI-Bullying factors of the BCBQ questionnaire were not normally distributed, thus we used appropriate estimators in the analyses.

To assess Bully-Victim status, we created groups from the Bullying and Victimization subscales with a cutoff of +1 standard deviation above the mean of the given scale. Individuals scoring above *M* + 1 SD on both bullying perpetration (>16.90) and victimization (>22.93) were classified as Bully-Victims (*n* = 25), while the remaining participants were categorized accordingly into Bullies (*n* = 11; above cutoff in perpetration, below cutoff in victimization), Victims (*n* = 29; below cutoff in perpetration, above cutoff in victimization), or Non-Involved groups (*n* = 224; below cutoff in both). We examined differences in AI-related bullying-like behavior across these groups using one-way ANOVA with a Welch correction and Games-Howell *post-hoc* tests.

To examine the hypothesized factors associated with bullying-like behavior toward conversational AI, we conducted a linear regression with AI-bullying as the dependent variable, and social anxiety, AI literacy components (Apply AI, Persuasion Literacy, and Emotion Regulation), prior bullying perpetration, and prior bullying victimization as predictors. We included the interaction between bullying and victimization to control for Bully-Victim status; additionally we controlled for sex (excluding individuals who selected ‘prefer not to say’), since the literature reports significant sex differences in bullying behaviors. We also controlled for AI use frequency as a continuous variable calculated from reported frequency across all platforms, since individuals who rarely or never use conversational AI compared to those who often do, have fewer opportunities to engage in such behavior. All terms were simultaneously added as one model. We report unstandardized coefficients to preserve the original unit, but also to make interpretability easier due to the interaction term in the analysis. Inspection of residual diagnostics indicated that the central portion of the residuals followed a normal distribution, with only minor deviations in the upper tail. Given the sample size, the model estimates can be considered robust to such deviations. The Durbin-Watson statistic (DW = 2.04, *p* = 0.747) indicated no autocorrelation among residuals. Potential influential observations were examined using Cook’s distance, with all values well below commonly accepted thresholds (maximum = 0.740), suggesting that no single observation unduly influenced the model. Overall, these diagnostics support the adequacy of the regression model.

Finally, to test the third hypothesis, we conducted a mediation analysis with interaction anxiety (SIAS) as predictor variable, AI-Bullying as outcome variable and victimization as the mediating variable. We used the diagonally weighted least squares (DWLS) estimator which is appropriate for non-normal distribution (Bandalos, 2014).

## 3 Results

We began by testing our first hypothesis regarding bullying group differences in CAI bullying-like behavior. We found a significant difference in AI bullying-like behavior across groups defined by prior bullying experiences (*F*(3, 31.5) = 20.6, *p* < 0.001, η_p_^2^ = 0.390, see Figure 1). In accordance with our predictions, participants in the Bully-Victim group were the most likely to show bullying-like behavior toward CAI (*M* = 14.92, SD = 5.56). Participants in the pure Bullying group scored lower than them (*M* = 11.64, SD = 4.13), but higher than the Non-involved (*M* = 7.04, SD = 2.60) and the Victim (*M* = 6.69, SD = 1.65) groups. The latter two did not differ from each other and were the least likely to engage in CAI bullying-like behavior. *Post hoc* tests are presented in Table 5.

**Figure 1:**
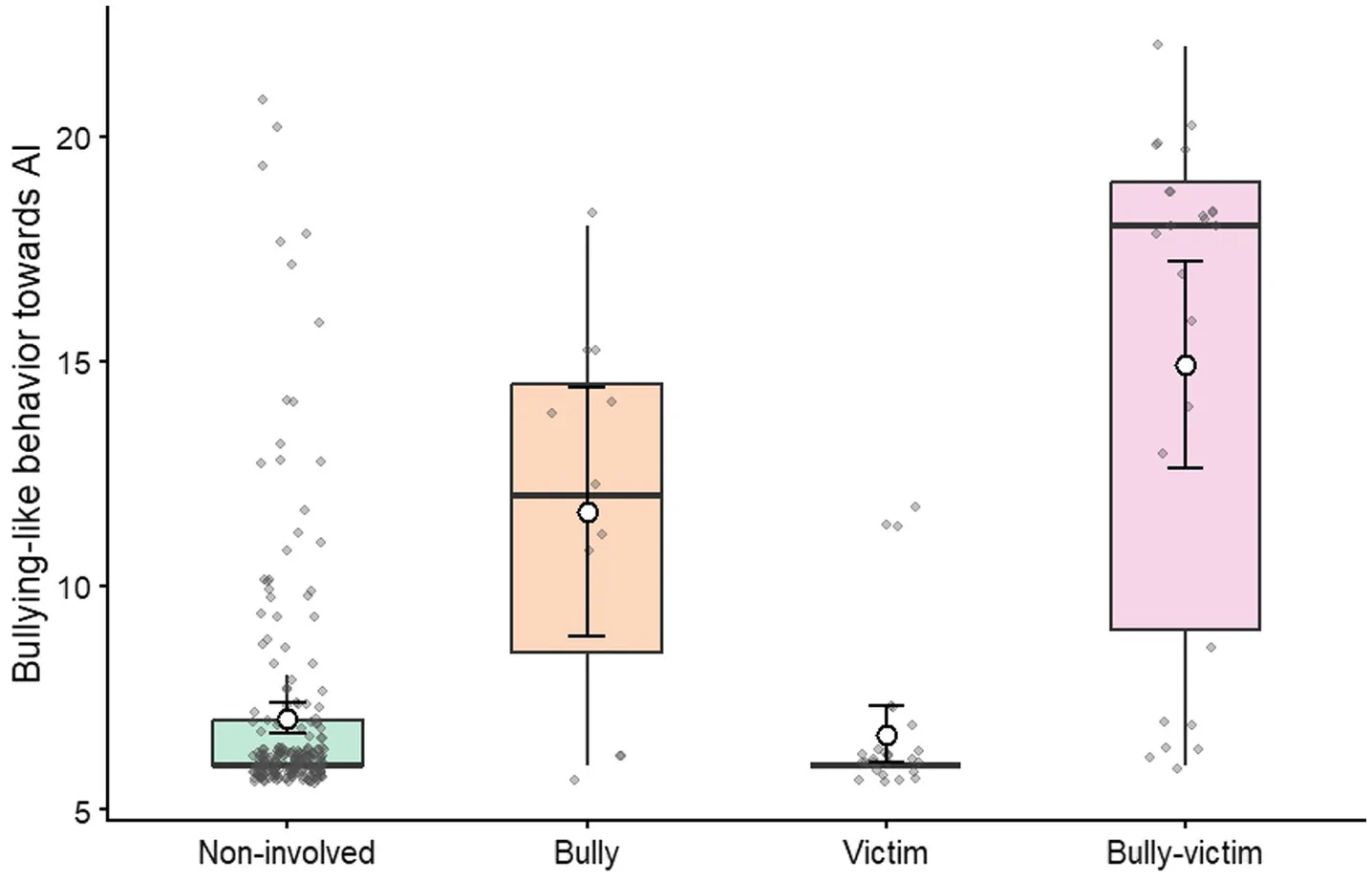
Bullying-like behavior toward AI in reflection of previous bullying and victimization experiences.

**Table 5:** *Post hoc* comparisons of AI-directed bullying-like behavior across bullying role groups.

Comparison	Mean Difference	SE	*t*	df	*p*
Non-involved - Bully-victim	−7.88	1.125	−7.002	25.18	< 0.001
Non-involved - Victim	0.351	0.352	0.996	48.23	0.753
Non-involved - Bully	−4.596	1.257	−3.656	10.39	0.018
Bully-victim - Victim	8.23	1.153	7.136	27.65	< 0.001
Bully-victim - Bully	3.284	1.669	1.967	25.54	0.227
Victim - Bully	−4.947	1.282	−3.858	11.23	0.012

*Post hoc* tests were performed using a Games-Howell correction.

Next, we tested our second hypothesis regarding the predictors of bullying-like behavior toward CAI. The regression model was significant (*F*(9, 274) = 27.5, *p* < 0.001, *R*^2^_adj_ = 0.457), explaining approximately 45.7% of the variance in bullying-like behavior toward conversational AI. The control variable of sex was a significant predictor, with males (*M* = 8.11, SE = 0.335) reporting higher levels of AI-directed bullying-like behavior compared to females (*M* = 7.19, SE = 0.210) (*b* = 0.924, SE = 0.377, *t* = 2.452, *p* = 0.015). AI use frequency was also a significant predictor (*b* = 0.181, SE = 0.043, *t* = 4.163, *p* < 0.001). Individuals reporting more frequent AI use expectedly reported higher levels of AI-directed bullying-like behavior; while infrequent users reported lower levels of bullying-like tendencies toward AI (*M* = 6.87, SE = 0.272), frequent users reported higher levels of these behaviors (*M* = 8.42, SE = 0.285).

Prior victimization showed a significant negative association with AI-directed bullying (*b* = −0.255, SE = 0.066, *t* = −3.852, *p* < 0.001). However, the interaction between bullying perpetration and victimization was also a strong and significant predictor (*b* = 0.022, SE = 0.005, *t* = 4.467, *p* < 0.001), indicating that the relationship between prior bullying behavior and AI-directed bullying-like behavior depends on levels of victimization (see Figure 2). Specifically, individuals who reported both high levels of perpetration and victimization exhibited the highest levels of bullying-like behavior toward conversational AI. Higher levels of victimization were associated with lower AI-directed bullying-like behavior in individuals with low prior bullying perpetration, and higher levels of AI bullying-like behavior in participants with high prior bullying perpetration.

**Figure 2:**
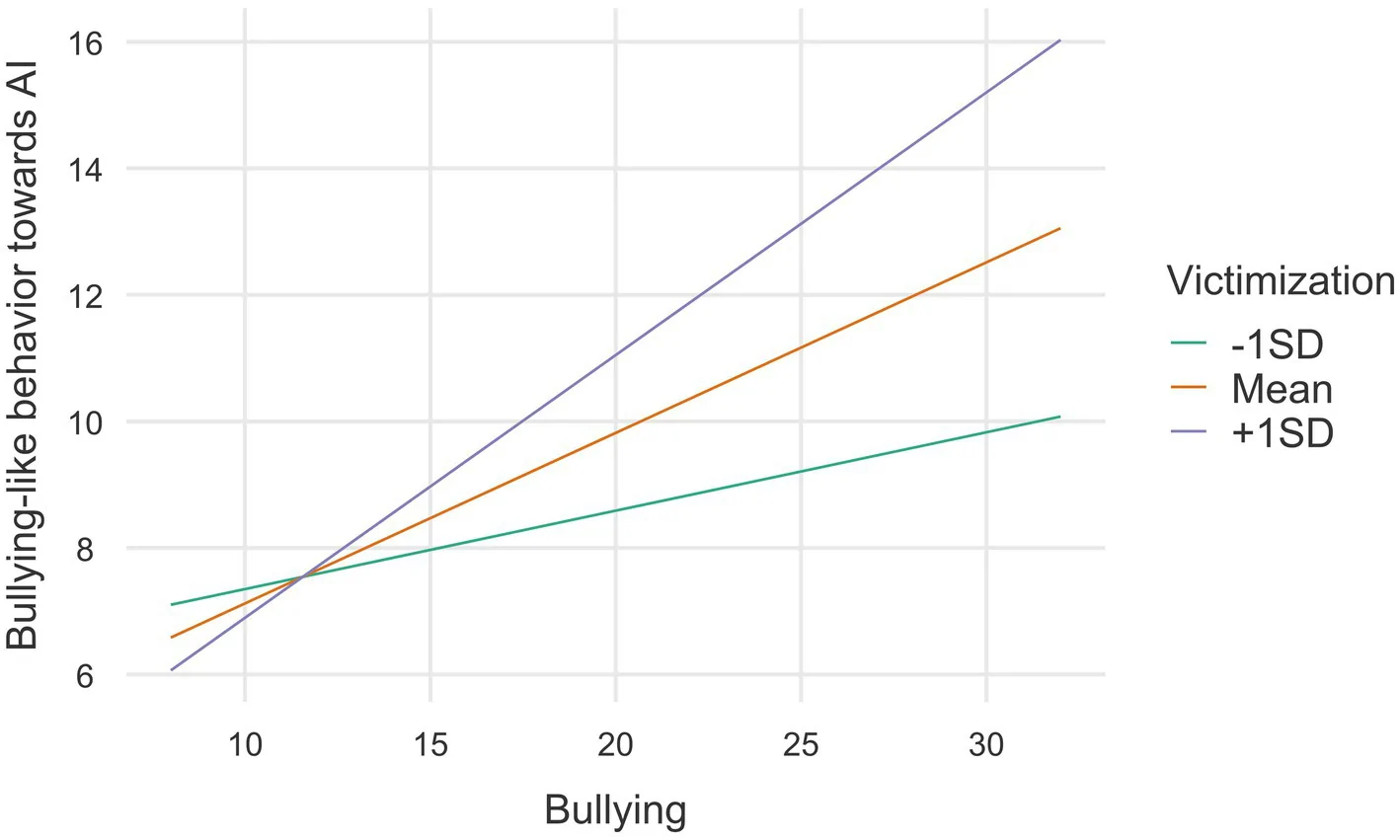
The effects of prior bullying and victimization on bullying-like behavior directed toward conversational AI.

Contrary to our hypothesis, we found no associations between AI-directed bullying and social anxiety (*b* = 0.060, SE = 0.034, *t* = 1.792, *p* = 0.074) or prior bullying perpetration (*b* = −0.087, SE = 0.118, *t* = −0.736, *p* = 0.462). None of the AI self-competency variables were significant predictors either, including Apply AI (*b* = −0.039, SE = 0.025, *t* = −1.559, *p* = 0.120), Persuasion Literacy (*b* = −0.069, SE = 0.066, *t* = −1.040, *p* = 0.299), and Emotion Regulation (*b* = 0.112, SE = 0.066, *t* = 1.704, *p* = 0.090).

Finally, we tested our third hypothesis whether victimhood could be an influencing factor in the relationship between social anxiety and bullying-like behavior toward CAI. Interaction anxiety was positively associated with victimhood (*β* = 0.314, *z* = 4.278, *p* < 0.001), meaning that individuals who reported higher social anxiety reported more experiences of bullying victimhood. Victimhood was also positively associated with bullying-like behaviors toward CAI (*β* = 0.327, *z* = 3.282, *p* = 0.001), which means that individuals who experienced being bullied, reported higher levels of bullying-like behavior toward AI. And although the direct effect of social anxiety on bullying-like behaviors toward CAI was not significant (*β* = 0.044; *z* = 0.573; *p* = 0.567), the indirect effect with the mediator factor of victimhood was significant (*β* = 0.103, *z* = 2.509, *p* = 0.012), indicating that individuals with higher interaction anxiety show bullying-like behavior toward CAI only if they are victimized in real life, in line with our hypothesis.

## 4 Discussion

Our study aimed to examine how prior experiences with bullying, social anxiety, and individual differences in AI literacy relate to bullying-like behavior directed toward CAI. We sought to clarify whether interpersonal social strategies calibrated for human-human interaction are transferred to artificial agents that are perceived as social. The results reveal two core findings: first, that bullying-like behavior toward CAI was strongly predicted by the interaction of perpetration and victimization, with bully-victims exhibiting the highest levels of AI-directed aggression; and second, that social anxiety was indirectly associated with CAI-directed bullying-like behavior through victimization experiences.

Consistent with the first hypothesis, individuals who self-reported as Bully-Victims, rather than ‘pure’ Bullies, exhibited the highest amount of bullying-like behavior toward AI. In line with this, bullying on its own was not a significant predictor in our regression model aimed to explain bullying-like behavior toward AI; instead, it was highest when both prior bullying and victimization were at high levels, while victimization in itself was a negative predictor, however, its effect is dependent on levels of prior perpetration. At low perpetration levels, the effect of victimization was negative, and at high perpetration, it was positive.

Contrary to our expectations, AI self-competency was not directly associated with AI-directed bullying-like behavior. The dominance of prior bullying perpetration and victimization as a predictor among factors like social anxiety and AI literacy supports the notion that bullying reflects a relatively stable social strategy (Chapell et al., 2006). This strategy generalizes across interactional contexts (Pabian and Vandebosch, 2021), including those involving artificial agents.

We found a significant effect of biological sex, with males reporting higher levels of AI-directed bullying-like behavior than females. This finding aligns with broader cyberbullying research (Bergmann and Baier, 2018) where males often report higher levels of psychological and sexualized online aggression, as they demonstrate stronger associations between moral disengagement and cyber-perpetration (Baltzidis, 2024). In the context of AI, this may manifest as males being more willing to enact dominance-oriented behaviors toward agents perceived as social targets but lacking agency.

Social anxiety was only indirectly associated with bullying-like behavior toward CAI through experiences of victimization. This suggests that socially anxious individuals are not more likely to show bullying-like behavior toward artificial agents per se, but that they are more likely to do so when they have experienced victimization in real-world social contexts. This pattern is consistent with previous research showing that social anxiety predicts bullying victimization (Deng et al., 2024), which may foster frustration, anger, and a desire to regain control, which can manifest as defensive or displaced aggression (Martin and Vieaux, 2015), particularly in contexts that are perceived as low-risk. As Conversational AI offers social cues sufficient to trigger dominance and evaluation-related processes, it simultaneously removes the risk of social rejection or punishment. In this sense, bullying-like behavior toward CAI may function as an outlet for unresolved distress associated with past victimization, rather than as a direct consequence of anxiety alone.

The centering of victimization across all analyses suggests that CAI-directed aggression may function as a form of defensive displacement. Individuals who have been victimized often experience anxiety, unresolved anger, and a perceived loss of social control (Broekhof et al., 2018; Guo et al., 2022). For these individuals, conversational AI might provide a unique social context where the target displays enough human-like cues to activate social-cognitive schemas but, from the perspective of the user, might lack the ability to retaliate or impose reputational costs. Bully-Victims might show the most extreme behavior because they possess both the drive (the need to reclaim dominance) and the tools (the behavioral repertoire of specific aggressive strategies) to use CAI as a regulatory outlet (Guy et al., 2019). This interpretation aligns with previous research showing that Bully-Victims exhibit higher emotional dysregulation and maladaptive coping compared to uninvolved individuals (Arató et al., 2020; Coelho and Romão, 2018). In contrast, ‘pure’ victims may lack the established strategies for dominance assertion (Salmivalli and Nieminen, 2002), explaining their lower levels of bullying-like behavior toward AI, whereas the non-significant result in the case of pure Bullies suggests that having aggressive tendencies is not sufficient to produce AI-directed bullying-like behavior in this context, as AI may not give them the social reward or status gain they seek (Van Dijk et al., 2017).

Our results align with evolutionary mismatch and anthropomorphism accounts. CAI systems display cues such as linguistic fluency, turn-taking, and responsiveness, which seem to activate social-cognitive mechanisms, including agency detection and dominance tracking (Guingrich and Graziano, 2024; Li et al., 2018; Lim et al., 2026; Sundar, 2020). However, if we view AI not only as a new technology, but indeed as a new social partner, our results have even wider implications; suggesting that users do not just transfer interpersonal patterns but adapt them to a new category of relational agent (Alqasir, 2025). As AI systems become integrated as companions and partners (Boyd and Markowitz, 2026), aggressive interactions may represent a failure of socio-affective alignment, a dynamic where the AI’s lack of reciprocity can reinforce specific, stable user tendencies that are becoming a core part of our technological social ecosystem (Guingrich and Graziano, 2024).

While our data is cross-sectional and not fit to establish causality, a critical question raised by these findings concerns whether expressing aggression toward a CAI system – which from the perspective of the user might not experience harm or distress – may serve a cathartic function, or whether it instead reinforces aggressive behavioral patterns that later generalize back to human targets. Early catharsis theories suggested that venting anger could reduce subsequent aggression; however, empirical research has consistently failed to support this notion (Bushman et al., 1999). Instead, expressing aggression, even against non-sentient or symbolic targets, tends to increase rather than decrease later aggressive behavior (Bushman, 2002). From a social-cognitive perspective, aggression is learned and reinforced through enactment: behaviors that successfully reduce tension or restore a sense of control become more likely to be repeated in the future (Bandura, 1978). In this sense, directing bullying-like behavior toward AI may function as a low-cost rehearsal of aggression, particularly for individuals who already possess these interpersonal strategies.

Research on displaced aggression and cyberbullying supports this concern. Studies show that when individuals cannot safely retaliate against the original source of frustration or threat, they may redirect aggression toward targets perceived as safe, anonymous, or unable to retaliate (Marcus-Newhall et al., 2000). Over time, such displaced aggression does not resolve underlying distress but instead strengthens hostile attribution biases and aggressive response tendencies. As such, repeated aggressive behavior toward targets like online avatars, game characters, or chatbots, may further desensitize individuals to norms of respect and empathy (Barlett et al., 2016; Lacković et al., 2023). This concern is also shown by philosophical arguments that cruelty toward non-sentient social agents might have effects on how sentient beings like humans and animals are treated (Coghlan et al., 2019). Applied to CAI, this suggests that treating AI as an acceptable object of abuse may legitimize aggression, increasing the likelihood that individuals will revert to similar behaviors in human-human interactions when situational constraints are reduced. Rather than providing a safe outlet, bullying-like behavior toward AI may – based on theoretical accounts of displaced aggression – therefore contribute to the maintenance and reinforcement of an aggressive cycle, particularly among individuals with prior victimization, social anxiety, or established bullying tendencies.

Previous studies regarding the emotional connections of AI use show a complex pattern. On one hand, online environments can be considered more safe and allow greater flexibility in self-presentation and more control than a face-to-face situation (Erwin et al., 2004), as has been shown with multiplayer video-game communities (Martončik and Lokša, 2016). Similarly, a conversation with AI can function as a form of low-pressure social practice or even emotional support and companionship, potentially alleviating loneliness (De Freitas et al., 2026; Hollis, 2026; Hu et al., 2023). This aligns with the concept of repertoire degradation (Niculescu, 2026), whereby repeated engagement with AI systems that provide frictionless validation may progressively narrow users’ emotional coping repertoire, replacing costly but productive strategies such as sublimation, humor, or anticipation with simpler externalization. However, for individuals with preexisting vulnerabilities, such as social anxiety and prior victimization, this trajectory represents a risk of AI becoming a relational context in which aggressive and avoidant strategies are continuously rehearsed and reinforced without the corrective feedback that human relationships provide. In line with this, increased engagement with AI chatbots has been associated with higher levels of emotional dependence and problematic use, particularly among individuals who rely on these systems for social or affective needs (Fang et al., 2025; Manoli et al., 2026; Pentina et al., 2023). Chu et al. (2025), analyzing conversations with social chatbots, found that chatbots can mirror and amplify user affect, producing emotional synchrony patterns that resemble human intimacy formation. In cases involving users with maladaptive coping styles, these dynamics devolved into toxic interaction patterns, including verbal abuse and self-harm ideation. Our research adds to this by highlighting a unique relational dynamic: AI might act as a regulatory tool for individuals caught in a maladaptive cycle of victimization and aggression. By providing a ‘safe’ target that activates social-cognitive schemas without the risk of social rejection, CAI may inadvertently reinforce the very coping strategies (dominance assertion and aggression) that maintain the individual’s interpersonal difficulties.

These findings carry direct implications for clinical practice. The use of AI as therapist has already raised significant concerns (Békés and Aafjes-van Doorn, 2026; Cooper et al., 2026; Khawaja and Bélisle-Pipon, 2023), and as chatbot use becomes widespread, clinicians should assess not only whether patients use AI systems, but how the relational dynamics of those interactions shape emotional regulation over time. The concept of liquidity illusion (Niculescu, 2026) is particularly relevant, as patients may present with articulate emotional vocabulary developed through chatbot interaction, yet show no genuine change in emotional regulation capacity or relational functioning. This concern is consistent with recent studies finding that users often experience emotional support from AI systems as validating and non-judgmental, which also carries the risk of over-reliance and incorrect advice (Giray, 2025; Jung et al., 2025). Recent research suggests that this might especially be relevant for neurodivergent populations such as individuals with autistic traits (Hollis, 2026; Suriano et al., 2026). For individuals with bullying histories and social anxiety, like those in our sample, the risk is that CAI interaction provides the appearance of emotional processing while reinforcing the very patterns that maintain distress.

### 4.1 Limitations and future directions

Several limitations should be noted. The study relied on self-report measures, which may be influenced by social desirability or recall bias, and participants mainly consisted of female university students. As AI-self competency slightly fell below the commonly accepted threshold of internal consistency, results involving this dimension should be interpreted with caution. Additionally, the bullying role groups had uneven sizes, thus results concerning them, especially the Bullies should be interpreted cautiously.

It is important to note that the present findings primarily reflect interactions with text-based CAI systems. Other modalities, such as voice assistants or embodied agents, provide additional social cues (e.g., tone of voice, physical presence) that may alter anthropomorphization and, consequently, the expression of dominance-related behaviors. Additionally, it is important to note that since artificial intelligence is a novel and rapidly evolving field, our findings may at the time of submission already be partially outdated.

Future research should adopt longitudinal and trajectory-based designs to track how emotional (dys)regulation evolves across sustained human-AI interaction, as the cross-sectional design of the present study cannot capture the temporal dynamics of these factors, nor establish causality. As bullying behavior in general exhibits sex-based differences, future studies should aim for more balanced samples in this regard.

Despite these limitations, the present study provides novel evidence that conversational AI functions as a meaningful social target for the expression of transferred interpersonal strategies. As AI systems become increasingly integrated into everyday life, understanding these dynamics will be essential for anticipating both the benefits and risks of human-AI interaction.

## Data Availability

The raw data supporting the conclusions of this article will be made available by the authors, without undue reservation.

## References

[ref1] Alqasir A. (2025). The relational shift: why we need “AI psychology” now as a core field. J. Psychol. AI 1:2573928. doi: 10.1080/29974100.2025.2573928

[ref2] Arató N. Zsidó A. N. Lénárd K. Lábadi B. (2020). Cybervictimization and cyberbullying: the role of socio-emotional skills. Front. Psych. 11:248. doi: 10.3389/fpsyt.2020.00248, 32346369 PMC7169421

[ref3] Baltzidis E. (2024). Gender differences in cyberbullying perpetration on Facebook: the role of empathy, callous unemotional traits, and moral disengagement. Psychol. Soc. Educ. 16, 53–62. doi: 10.21071/pse.v16i3.16997

[ref4] Bandalos D. L. (2014). Relative performance of categorical diagonally weighted least squares and robust maximum likelihood estimation. Struct. Equ. Model. 21, 102–116. doi: 10.1080/10705511.2014.859510

[ref5] Bandura A. (1978). Social learning theory of aggression. J. Commun. 28, 12–29. doi: 10.1111/j.1460-2466.1978.tb01621.x 690254

[ref6] Barlett C. P. Gentile D. A. Chew C. (2016). Predicting cyberbullying from anonymity. Psychol. Pop. Media Cult. 5, 171–180. doi: 10.1037/ppm0000055

[ref7] Békés V. Aafjes-van Doorn K. (2026). Who wants to have an AI therapist? Acceptance of using artificial intelligence for mental health interventions among clinicians, patients and the general community. Clin. Psychol. Psychother. 33:e70220. doi: 10.1002/cpp.70220, 41578705 PMC12831102

[ref8] Bergmann M. Baier D. (2018). Prevalence and correlates of cyberbullying perpetration. Findings from a German representative student survey. Int. J. Environ. Res. Public Health 15:274. doi: 10.3390/ijerph15020274, 29415426 PMC5858343

[ref9] Bigman Y. E. Briker R. Langer M. (2026). Human–AI interaction research needs to be embedded in psychological theory. Nat. Rev. Psychol. 5, 233–234. doi: 10.1038/s44159-026-00551-4

[ref10] Boyd R. L. Markowitz D. M. (2026). Artificial intelligence and the psychology of human connection. Perspect. Psychol. Sci. 21, 192–220. doi: 10.1177/17456916251404394, 41608879 PMC12960742

[ref11] Brandtzaeg P. B. Følstad A. (2017). “Why people use Chatbots,” in Internet Science, eds. Kompatsiaris I. Cave J. Satsiou A. Carle G. Passani A. Kontopoulos E. . (Oslo, Norway: Springer International Publishing), 377–392.

[ref12] Broekhof E. Bos M. G. N. Camodeca M. Rieffe C. (2018). Longitudinal associations between bullying and emotions in deaf and hard of hearing adolescents. J. Deaf Stud. Deaf Educ. 23, 17–27. doi: 10.1093/deafed/enx036, 28977584

[ref13] Bushman B. J. (2002). Does venting anger feed or extinguish the flame? Catharsis, rumination, distraction, anger, and aggressive responding. Personal. Soc. Psychol. Bull. 28, 724–731. doi: 10.1177/0146167202289002

[ref14] Bushman B. J. Baumeister R. F. Stack A. D. (1999). Catharsis, aggression, and persuasive influence: self-fulfilling or self-defeating prophecies? J. Pers. Soc. Psychol. 76, 367–376. doi: 10.1037/0022-3514.76.3.367, 10101875

[ref15] Carolus A. Augustin Y. Markus A. Wienrich C. (2023a). Digital interaction literacy model – conceptualizing competencies for literate interactions with voice-based AI systems. Comput. Educ. Artif. Intell. 4:100114. doi: 10.1016/j.caeai.2022.100114

[ref16] Carolus A. Koch M. J. Straka S. Latoschik M. E. Wienrich C. (2023b). MAILS - Meta AI literacy scale: development and testing of an AI literacy questionnaire based on well-founded competency models and psychological change- and meta-competencies. Comput. Human Behav. 1:100014. doi: 10.1016/j.chbah.2023.100014

[ref17] Chapell M. S. Hasselman S. L. Kitchin T. Lomon S. N. Maclver K. W. Sarullo P. L. (2006). Bullying in elementary school, high school, and college. Adolescence 41, 633–648. 17240771

[ref18] Chu M. D. Gerard P. Pawar K. Bickham C. Lerman K. (2025). Illusions of Intimacy: how Emotional Dynamics Shape human-AI Relationships. preprint arXiv:2505.11649

[ref19] Coelho V. A. Romão A. M. (2018). The relation between social anxiety, social withdrawal and (cyber)bullying roles: a multilevel analysis. Comput. Hum. Behav. 86, 218–226. doi: 10.1016/j.chb.2018.04.048

[ref20] Coelho V. A. Sousa V. (2020). Bullying and cyberbullying behaviors questionnaire: validation of a short form. Int. J. Sch. Educ. Psychol. 8, 3–10. doi: 10.1080/21683603.2018.1522282

[ref21] Coghlan S. Vetere F. Waycott J. Barbosa Neves B. (2019). Could social robots make us kinder or crueller to humans and animals? Int. J. Soc. Robot. 11, 741–751. doi: 10.1007/s12369-019-00583-2

[ref22] Cooper N. Guridi J. A. Hwang A. H.-C. Kolko B. McGinty E. E. Yang Q. (2026) Framing responsible design of AI for mental well-being: AI as primary care, nutritional supplement, or yoga instructor? Proceedings of the 2026 CHI Conference on Human Factors in Computing Systems New York, NY. 1–16

[ref23] Cosmides L. Tooby J. (1994). “Origins of domain specificity: the evolution of functional organization,” in Mapping the Mind, eds. Hirschfeld L. A. Gelman S. A.. 1st ed (Cambridge: Cambridge University Press), 85–116.

[ref24] De Freitas J. Oğuz-Uğuralp Z. Uğuralp A. K. Puntoni S. (2026). AI companions reduce loneliness. J. Consum. Res. 52, 1126–1148. doi: 10.2139/ssrn.4893097

[ref25] Deng J. Liu J. Luo J. Pi Y. Pan J. Fu Z. . (2024). Social anxiety and bullying victimization: a three-level meta-analysis of cross-sectional and longitudinal studies. Child Abuse Negl. 157:107052. doi: 10.1016/j.chiabu.2024.107052, 39306940

[ref26] Epley N. Waytz A. Cacioppo J. T. (2007). On seeing human: a three-factor theory of anthropomorphism. Psychol. Rev. 114, 864–886. doi: 10.1037/0033-295X.114.4.864, 17907867

[ref27] Erwin B. A. Turk C. L. Heimberg R. G. Fresco D. M. Hantula D. A. (2004). The internet: home to a severe population of individuals with social anxiety disorder? J. Anxiety Disord. 18, 629–646. doi: 10.1016/j.janxdis.2003.08.002, 15275943

[ref28] Fang C. M. Liu A. R. Danry V. Lee E. Chan S. W. T. Pataranutaporn P. . (2025). How AI and human Behaviors Shape Psychosocial Effects of Extended Chatbot use: a Longitudinal Randomized Controlled Study. [preprint on Arxiv]. doi: 10.48550/arXiv.2503.17473

[ref29] Faul F. Erdfelder E. Lang A.-G. Buchner A. (2007). G*power 3: a flexible statistical power analysis program for the social, behavioral, and biomedical sciences. Behav. Res. Methods 39, 175–191. doi: 10.3758/BF03193146, 17695343

[ref30] Gambino A. Fox J. Ratan R. (2020). Building a stronger CASA: extending the computers are social actors paradigm. Hum. Mach. Commun. 1, 71–86. doi: 10.30658/hmc.1.5

[ref31] Giray L. (2025). Cases of using ChatGPT as a mental health and psychological support tool. J. Consum. Health Internet 29, 29–48. doi: 10.1080/15398285.2024.2442374

[ref32] Guingrich R. E. Graziano M. S. A. (2024). Ascribing consciousness to artificial intelligence: human-AI interaction and its carry-over effects on human-human interaction. Front. Psychol. 15:1322781. doi: 10.3389/fpsyg.2024.1322781, 38605842 PMC11008604

[ref33] Gunkel D. J. (2023). Person, Thing, Robot: A Moral and Legal Ontology for the 21st Century and beyond. Cambridge, MA, USA: The MIT Press.

[ref34] Guo Y. Tan X. Zhu Q. (2022). Chains of tragedy: the impact of bullying victimization on mental health through mediating role of aggressive behavior and perceived social support. Front. Psychol. 13:988003. doi: 10.3389/fpsyg.2022.988003, 36425817 PMC9679517

[ref35] Guy A. Lee K. Wolke D. (2019). Comparisons between adolescent bullies, victims, and bully-victims on perceived popularity, social impact, and social preference. Front. Psych. 10:868. doi: 10.3389/fpsyt.2019.00868, 31824358 PMC6883422

[ref36] Heimberg R. G. Brozovich F. A. Rapee R. M. (2014). “A cognitive-behavioral model of social anxiety disorder,” in Social Anxiety, Eds. Stefan G. Hofmann, Patricia M. DiBartolo. (Cambridge, MA: Academic Press, Elsevier), 705–728.

[ref37] Hollis A. (2026) “Tinged with heartbreak”: an ethnographic account of navigating autistic loneliness and the fragile promise of AI companionship Proceedings of the 2026 CHI Conference on Human Factors in Computing Systems. New York, NY, United States. 1–13

[ref38] Hortensius R. Cross E. S. (2018). From automata to animate beings: the scope and limits of attributing socialness to artificial agents. Ann. N. Y. Acad. Sci. 1426, 93–110. doi: 10.1111/nyas.13727, 29749634

[ref39] Hu B. Mao Y. Kim K. J. (2023). How social anxiety leads to problematic use of conversational AI: the roles of loneliness, rumination, and mind perception. Comput. Hum. Behav. 145:107760. doi: 10.1016/j.chb.2023.107760

[ref40] Ige T. Adewale S. (2022). AI powered anti-cyber bullying system using machine learning algorithm of multinomial naïve bayes and optimized linear support vector machine. Int. J. Adv. Comput. Sci. Appl. 13. doi: 10.14569/IJACSA.2022.0130502

[ref41] International Test Commission (2018). The ITC Guidelines for Translating and Adapting Tests. Second Edn. Int. J. Testing, 18, 101–134. doi: 10.1080/15305058.2017.1398166

[ref42] Jung K. Lee G. Huang Y. Chen Y. (2025). “I’ve talked to ChatGPT about my issues last night.”: examining mental health conversations with large language models through Reddit analysis. Proc. ACM Hum.-Comput. Interact. 9, 1–25. doi: 10.1145/3757537

[ref43] Juvonen J. Graham S. Schuster M. A. (2003). Bullying among young adolescents: the strong, the weak, and the troubled. Pediatrics 112, 1231–1237. doi: 10.1542/peds.112.6.1231, 14654590

[ref44] Kahn P. H. Reichert A. L. Gary H. E. Kanda T. Ishiguro H. Shen S. . (2011) The new ontological category hypothesis in human-robot Interaction Proceedings of the 6th International Conference on Human-Robot Interaction New York, NY, United States: Association for Computing Machinery. 159–160

[ref45] Karnaze M. M. Bloss C. S. (2026). Six reasons to study emotional support from conversational artificial intelligence. Nat. Hum. Behav. 10, 635–638. doi: 10.1038/s41562-026-02412-9, 41688600

[ref46] Khawaja Z. Bélisle-Pipon J.-C. (2023). Your robot therapist is not your therapist: understanding the role of AI-powered mental health chatbots. Front. Digital Health 5:1278186. doi: 10.3389/fdgth.2023.1278186, 38026836 PMC10663264

[ref47] Kim J. Im I. (2023). Anthropomorphic response: understanding interactions between humans and artificial intelligence agents. Comput. Hum. Behav. 139:107512. doi: 10.1016/j.chb.2022.107512

[ref48] Kirk H. R. Gabriel I. Summerfield C. Vidgen B. Hale S. A. (2025). Why human–AI relationships need socioaffective alignment. Humanit. Soc. Sci. Commun. 12:728. doi: 10.1057/s41599-025-04532-5

[ref49] Lacković D. Lotar Rihtarić M. Vrselja I. (2023). Perceived anonymity and cyberbullying: what happens when there is a lack of social influence? Hrvat. Rev. Rehabil. Istraz. 59, 31–42. doi: 10.31299/hrri.59.2.3

[ref50] Li N. P. Van Vugt M. Colarelli S. M. (2018). The evolutionary mismatch hypothesis: implications for psychological science. Curr. Dir. Psychol. Sci. 27, 38–44. doi: 10.1177/0963721417731378

[ref51] Lim A. J. Yong J. C. Tan E. (2026). Artificial intelligence, fundamental motives, and evolutionary mismatch. Evol. Behav. Sci. 20, 53–74. doi: 10.1037/ebs0000379

[ref52] Long D. Magerko B. (2020) What is AI literacy? Competencies and design considerations. Proceedings of the 2020 CHI Conference on Human Factors in Computing Systems. New York, NY, United States. 1–16

[ref53] Maeda T. (2025). Walkthrough of Anthropomorphic Features in AI Assistant Tools (Version 1). [Arxiv preprint] doi: 10.48550/arXiv.2502.16345

[ref54] Manoli A. Pauketat J. V. T. Anthis J. R. (2025) The AI double standard: humans judge all AIs for the actions of one. Proceedings of the ACM on Human-Computer Interaction 9, 1–24. doi: 10.1145/3711083

[ref55] Manoli A. Pauketat J. V. T. Ladak A. Noh H. Hwang A. H.-C. Anthis J. R. (2026) Digital companionship: overlapping uses of AI companions and AI assistants. Proceedings of the 2026 CHI Conference on Human Factors in Computing Systems. 9, 1–25. doi: 10.1145/3772318.3791331

[ref56] Marcus-Newhall A. Pedersen W. C. Carlson M. Miller N. (2000). Displaced aggression is alive and well: a meta-analytic review. J. Pers. Soc. Psychol. 78, 670–689. doi: 10.1037/0022-3514.78.4.670, 10794373

[ref57] Martin R. C. Vieaux L. E. (2015). “The digital rage: how anger is expressed online,” in The Psychology of Social Networking, eds. Riva G. Wiederhold B. K. Cipresso P. (Warsaw, Poland: De Gruyter Open Poland), 117–127.

[ref58] Martončik M. Lokša J. (2016). Do world of Warcraft (MMORPG) players experience less loneliness and social anxiety in online world (virtual environment) than in real world (offline)? Comput. Hum. Behav. 56, 127–134. doi: 10.1016/j.chb.2015.11.035

[ref59] Morgante E. Susinna C. Culicetto L. Quartarone A. Lo Buono V. (2024). Is it possible for people to develop a sense of empathy toward humanoid robots and establish meaningful relationships with them? Front. Psychol. 15:1391832. doi: 10.3389/fpsyg.2024.1391832, 39188868 PMC11346246

[ref60] Nass C. Steuer J. Tauber E. R. (1994). Computers are social actors. In Proceedings of the SIGCHI conference on Human factors in computing systems, Boston, Massachusetts, USA. 72–78.

[ref61] Navarro R. Yubero S. Larrañaga E. (2015). Psychosocial risk factors for involvement in bullying behaviors: empirical comparison between cyberbullying and social bullying victims and bullies. Sch. Ment. Health 7, 235–248. doi: 10.1007/s12310-015-9157-9

[ref62] Niculescu L. (2026). When the market asks no price: AI chatbot interaction as psychic market disruption. Front. Psych. 17:1812464. doi: 10.3389/fpsyt.2026.1812464, 42158146 PMC13180914

[ref63] Pabian S. Vandebosch H. (2021). Perceived long-term outcomes of early traditional and cyberbullying victimization among emerging adults. J. Youth Stud. 24, 91–109. doi: 10.1080/13676261.2019.1695764

[ref64] Pentina I. Hancock T. Xie T. (2023). Exploring relationship development with social chatbots: a mixed-method study of replika. Comput. Hum. Behav. 140:107600. doi: 10.1016/j.chb.2022.107600

[ref65] Peters L. Sunderland M. Andrews G. Rapee R. M. Mattick R. P. (2012). Development of a short form social interaction anxiety (SIAS) and social phobia scale (SPS) using nonparametric item response theory: the SIAS-6 and the SPS-6. Psychol. Assess. 24, 66–76. doi: 10.1037/a0024544, 21744971

[ref66] Salmivalli C. Nieminen E. (2002). Proactive and reactive aggression among school bullies, victims, and bully-victims. Aggress. Behav. 28, 30–44. doi: 10.1002/ab.90004

[ref67] Severson R. L. Carlson S. M. (2010). Behaving as or behaving as if? Children’s conceptions of personified robots and the emergence of a new ontological category. Neural Netw. 23, 1099–1103. doi: 10.1016/j.neunet.2010.08.014, 20851571

[ref68] Siegel R. S. La Greca A. M. Harrison H. M. (2009). Peer victimization and social anxiety in adolescents: prospective and reciprocal relationships. J. Youth Adolesc. 38, 1096–1109. doi: 10.1007/s10964-009-9392-1, 19636774

[ref69] Sundar S. S. (2020). Rise of machine agency: a framework for studying the psychology of human–AI interaction (HAII). J. Comput. Mediat. Commun. 25, 74–88. doi: 10.1093/jcmc/zmz026

[ref70] Suriano R. Plebe A. Furfari V. Fabio R. A. (2026). Emotional and cognitive factors in the relationship between autistic traits and emotional attachment to AI chatbots. Technol. Mind Behav. 7, 32–42. doi: 10.1037/tmb0000182

[ref71] Swearer S. M. Song S. Y. Cary P. T. Eagle J. W. Mickelson W. T. (2001). Psychosocial correlates in bullying and victimization: the relationship between depression, anxiety, and bully/victim status. J. Emot. Abus. 2, 95–121. doi: 10.1300/J135v02n02_07, 41340520

[ref72] Tomasello M. Carpenter M. (2007). Shared intentionality. Dev. Sci. 10, 121–125. doi: 10.1111/j.1467-7687.2007.00573.x, 17181709

[ref73] Van Dijk A. Poorthuis A. M. G. Malti T. (2017). Psychological processes in young bullies versus bully-victims. Aggress. Behav. 43, 430–439. doi: 10.1002/ab.21701, 28181256 PMC5573968

[ref74] Volk A. A. Dane A. V. Al-Jbouri E. (2022). Is adolescent bullying an evolutionary adaptation? A 10-year review. Educ. Psychol. Rev. 34, 2351–2378. doi: 10.1007/s10648-022-09703-3

[ref75] Weisman K. (2022) Extraordinary entities: insights into folk ontology from studies of lay people’s beliefs about robots. Proceedings of the 44th Annual Conference of the Cognitive Science Society Available online at: https://escholarship.org/uc/item/23p9h07t

